# Odynophagie als erstes Symptom bei Affenpocken-Infektion

**DOI:** 10.1007/s00106-023-01282-1

**Published:** 2023-03-31

**Authors:** Nadja Schröder, Juliane Buth, Ingo Drexler, Ortwin Adams, Inga Tometten, Maximilian Seidl, Christian Rubbert, Jörg Schipper, Julia Kristin

**Affiliations:** 1 Department of Oto-Rhino-Laryngology, Head and Neck Surgery, Düsseldorf University Hospital, Moorenstraße 5, 40225 Düsseldorf, Deutschland; 2 Institute of Pathology, Düsseldorf University Hospital, Düsseldorf, Deutschland; 3 Institute of Virology, Medical Faculty, Düsseldorf University Hospital, Heinrich-Heine University, Düsseldorf, Deutschland; 4 Department of Diagnostic and Interventional Radiology, Düsseldorf University Hospital, Düsseldorf, Deutschland

**Keywords:** Tonsillitis, Mpox, Lymphadenopathie, Orale Ulzeration, Tonsillektomie, Tonsillitis, Orthopoxvirus, Lymphadenopathy, Oral lesion, Tonsillectomy

## Abstract

Ein 50-jähriger Patient stellte sich mit Odynophagie und nächtlicher Dyspnoe bei einer bestätigten Affenpocken-Infektion vor. Klinisch zeigten sich eine Läsion am Zungenrand ohne Hautbefall sowie eine fibrinbelegte Tonsille mit Weichgaumenasymmetrie nach rechts. Aufgrund eines in der Computertomographie(CT)-Bildgebung gesicherten Abszesses erfolgte eine Tonsillektomie à chaud. Mittels Pan-Orthopox-spezifischer Polymerasekettenreaktion (PCR) wurde die Infektion auch im Tonsillengewebe bestätigt. Isolierte orale Befunde können eine Affenpocken-Infektion darstellen und stellen bei Risikopatienten eine aktuell wichtige Differenzialdiagnose dar.

## Abstract (en)

A 50-year-old patient with confirmed monkeypox infection presented with odynophagia and nocturnal dyspnea. Clinically, there was a lesion on the tongue without any skin lesions and fibrinous plaques on the right tonsil with asymmetry of the palatoglossal arch. Due to a suggested abscess in the CT scan, a tonsillectomy à chaud was performed. By pan-orthopox-specific polymerase chain reaction (PCR) the monkeypox infection was also confirmed in the tonsil tissue. Isolated oral findings may represent a monkeypox infection and should be considered as a currently important differential diagnosis, especially for patients at risks.

## Anamnese

Ein 50-jähriger Mann, der derzeit in den Niederlanden wohnt, stellte sich in unserer Notaufnahme mit Odynophagie und nächtlicher Dyspnoe vor. Der Patient klagte über anhaltende Halsschmerzen und Odynophagie seit neun Tagen. Der Hausarzt leitete bei Auftreten von Fieber drei Tage nach Beginn der Halsschmerzen eine Therapie mit Amoxicillin 1000 mg dreimal täglich und Mukolytika ein. Weitere zwei Tage später bemerkte der Patient eine Läsion auf der Zunge, woraufhin er sich erneut bei seinem Hausarzt in den Niederlanden vorstellte. Von der Läsion wurde ein Abstrich entnommen und mittels PCR eine Affenpocken-Infektion diagnostiziert. Wie die meisten Patienten, die derzeit von der Infektion betroffen sind, gehört dieser Patient zu der Risikogruppe, zu der auch Männer gehören, die Sex mit Männern haben (MSM). Wegen anhaltender Halsschmerzen über neun Tage mit zunehmender Odynophagie und nächtlicher Dyspnoe stellte sich der Patient in unserer Notaufnahme vor.

## Untersuchung

Aufgrund der bekannten Affenpocken-Infektion wurde die HNO-Untersuchung bereits in voller persönlicher Schutzausrüstung, bestehend aus Gesichtsschutz, Schutzkittel, OP-Haube, Gesichtsmaske und Untersuchungshandschuhen, durchgeführt.

### HNO-Untersuchung.

An der rechten Tonsille fanden sich Fibrinbeläge sowie eine Rötung und Asymmetrie zugunsten des rechten Gaumenbogens. Klinisch bestand der Verdacht auf einen Peritonsillarabszess. Abgesehen von der bekannten Läsion an der Zungenspitze wurden keine weiteren oralen Läsionen festgestellt. Die flexible Endoskopie ergab keine weiteren Läsionen im Bereich des Pharynx oder Larynx.

### Körperliche Untersuchung.

Eine vollständige körperliche Untersuchung wurde von den Kollegen der Medizinischen Klinik durchgeführt. Es ergaben sich keine weiteren pathologischen Befunde, insbesondere keine weiteren Hautveränderungen, die für eine Affenpocken-Infektion verdächtig sind.

## Diagnostische Ergebnisse

### Laborbefunde.

Die Blutuntersuchung ergab ein mäßig erhöhtes C‑reaktives Protein (CRP) von 3,5 mg/dl (< 0,5 mg/dl) bei einer normalen Leukozytenzahl. Die übrigen Laborparameter wie Leberwerte und Nierenwerte waren unauffällig.

### Radiologische Befunde.

**Figure 1:**
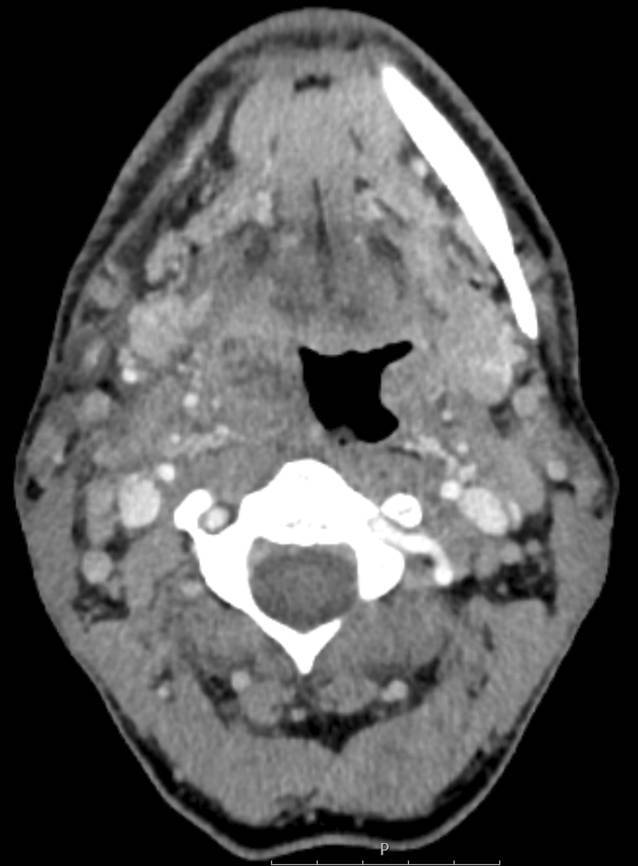

Die kontrastverstärkte Computertomographie (CT) des Halses zeigte eine vergrößerte rechte Gaumenmandel mit multiplen, teilweise konfluierenden, hypodensen Einschlüssen (Abb. 1). Darüber hinaus wurde eine ausgeprägte bizervikale Lymphadenopathie festgestellt.

## Therapie und Verlauf

Aufgrund der ausgeprägten und progredienten Symptomatik trotz oraler Antibiose und Analgesie, des klinischen Untersuchungsbefundes und des CT-Befundes entschieden wir uns für eine Tonsillektomie rechts. Intraoperativ war die rechte Tonsille mit weißen Belägen überzogen, der rechte Gaumenbogen war gerötet und vorstehend. Das Tonsillengewebe erschien klinisch sehr verhärtet und nekrotisch ähnlich zu Gewebeveränderungen, die bei infektiöser Mononukleose oder einem Peritonsillarabszess anderer Ursache auftreten. Als die Tonsille auf dem Operationstisch angeschnitten wurde, trat eine kleine Menge Eiter aus.

### Histologische Untersuchung.

**Figure 2:**
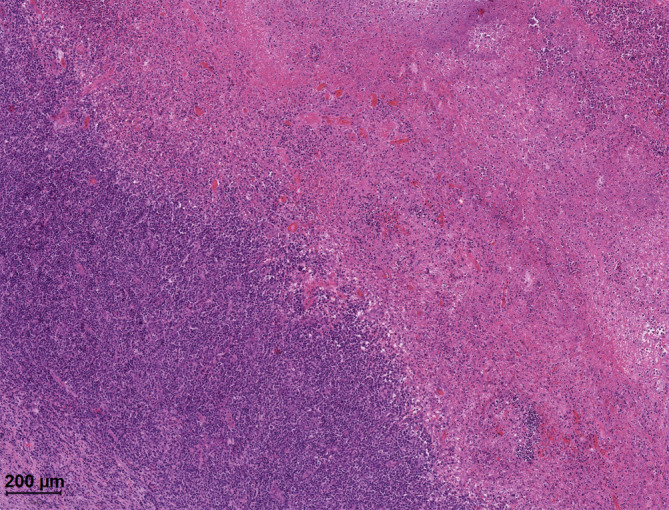

Das Tonsillektomiepräparat zeigte multifokale, kryptenassoziierte nekrotische Bereiche mit zahlreichen Neutrophilen. In der erhaltenen lymphatischen Pulpa waren wenige sekundäre lymphatische Follikel sichtbar, die parakortikalen/interfollikulären Bereiche waren erweitert und beinhalteten einige Immunoblasten (Abb. 2).

### Virologische Analyse.

Das Tonsillengewebe wurde homogenisiert, lysiert und mit Viral-Lysis-Buffer inaktiviert. Nach Extraktion und Reinigung wurde die aus dem Tonsillengewebe gewonnene DNA einer Pan-Orthopox-spezifischen Taqman-PCR unterzogen, bei der neben anderen Orthopoxviren verschiedene Stämme des Affenpockenvirus nachgewiesen wurden. Positive PCR-Ergebnisse bestätigten die Pockenvirusinfektion.

### Mikrobiologische Untersuchung.

Im Tonsillengewebe wurden Keime der oralen und pharyngealen Flora sowie eine anaerobe Mischflora nachgewiesen.

Am Tag der Krankenhausaufnahme begannen wir mit einer intravenösen Antibiotikatherapie mit Ampicillin/Sulbactam 2 g/1 g dreimal täglich und Metronidazol 500 mg zweimal täglich und verabreichten sie über insgesamt fünf Tage. Postoperativ besserten sich die Symptome des Patienten zeitnah, und der CRP-Wert im Blut sank auf normale Werte. In Anbetracht der bereits erfolgten oralen Antibiotikatherapie beschlossen wir, die Antibiotikatherapie nach insgesamt 11 Tagen abzusetzen. Der Patient wurde fünf Tage nach der Operation in die häusliche Quarantäne entlassen.

## Diskussion

Die Affenpocken-Infektion ist eine zoonotische Erkrankung, die durch das Affenpockenvirus verursacht wird. Das Virus wurde erstmals 1958 bei Affen entdeckt. Seit dem ersten Fall beim Menschen im Jahr 1970 sind Affenpocken-Infektionen in der Demokratischen Republik Kongo und in ganz Zentral- und Westafrika endemisch geworden. Der erste Ausbruch außerhalb Afrikas war 2003 in den USA. Es wird angenommen, dass dieser Ausbruch und die folgenden zwischen 2018 und 2021 auf eine Übertragung von Tier zu Mensch zurückzuführen sind [1]. Der derzeitige globale Ausbruch im Jahr 2022 ist der größte und am weitesten verbreitete Ausbruch von Affenpocken, der jemals beobachtet wurde. Etwa 75 % der in Europa festgestellten Fälle weisen eine ungewöhnlich hohe Häufigkeit der Übertragung von Mensch zu Mensch auf [8]. Die Übertragung kann einerseits direkt durch Kontakt mit infektiösem Hautausschlag, Körperflüssigkeiten oder Aerosolexposition über die Atemwege oder andererseits indirekt durch Kontakt mit kontaminierten Oberflächen erfolgen. Nach einer Inkubationszeit von 5 bis 21 Tagen wird ein Prodromalstadium mit Fieber, Schüttelfrost, Kopfschmerzen, Myalgien und Rückenschmerzen beschrieben, das 1 bis 3 Tage andauert. Darauf folgt in der Regel ein makulopapulöses und monomorphes Exanthem der Haut und/oder der Schleimhäute, typischerweise an der Inokulationsstelle (Anus, Genitalien). Eine ausgeprägte Lymphadenopathie ist ein charakteristisches Merkmal der Affenpocken-Infektion [3, 4, 6]. Derzeit sind in Deutschland 3556 Fälle gemeldet, darunter nur 17 weibliche [7]. Die zunehmende Zahl von Affenpocken-Infektionen erfordert die Aufmerksamkeit aller medizinischer Disziplinen. Wir stellen hier einen der zahlreichen Fälle in Deutschland vor, der atypische Symptome und Krankheitsverlauf aufwies.

Unser Patient zeigte als erstes Symptom Halsschmerzen und bis auf Fieber über einen Tag keine weiteren Manifestationen des Prodromalstadiums der Affenpocken-Infektion. Aufgrund der gezeigten Symptome war zu diesem Zeitpunkt von einer durch typische Erreger verursachten Tonsillitis auszugehen. Eine einzelne Läsion an der Zungenspitze ohne Hautausschlag oder Pocken am Anus bzw. den Genitalien trat erst fünf Tage später auf. Von solchen atypischen Fällen wurde nur selten berichtet. Bisher publizierte Fälle mit oralen Läsionen an den Tonsillen [5] oder einem retropharyngealen Abszess als Komplikation der Affenpocken-Infektion [4] entwickelten im Frühstadium immer den typischen Hautausschlag. In unserem Fall zeigte sich bis 12 Tage nach Beginn der Halsschmerzen außer der Zungenläsion kein weiteres Exanthem. Am Tag der Krankenhausentlassung erschien eine einzelne Pocke am Finger des Patienten. Dies ist eine erhebliche Verzögerung im Vergleich zum normalen Auftreten der Hautbefunde (2–3 Tage nach Fieber) [6]. Daher ist bei der Diagnose einer akuten Tonsillitis mit anderen Läsionen in der Mundhöhle höchste Aufmerksamkeit geboten, insbesondere bei Risikopatienten wie Männern, die Sex mit Männern (MSM) und mit mehreren Sexualpartnern haben.

Als Differenzialdiagnose wurde eine Affenpocken-Infektion mit zufälliger eitriger Tonsillitis vermutet. Die histologische Untersuchung des Präparats zeigte jedoch große, kryptenassoziierte Nekrosen in der Tonsille, was bei einer bakteriellen Tonsillitis nicht der Fall ist. Außerdem gab es nur eine geringe Dichte an sekundären lymphatischen Follikeln sowie einen erweiterten Parakortex mit einigen Immunoblasten, was auf eine geringere humorale und mehr zelluläre Immunantwort hindeutet. Ähnliche Befunde werden für EBV-assoziierte Tonsillitis berichtet [2]. In unserem Fall fanden wir keine Hodgkin-ähnlichen histologischen Merkmale. Schließlich wurde im chirurgischen Präparat Orthopox-Virus-DNA nachgewiesen, während sich mikrobiologisch nur Keime der Mund- und Rachenflora und eine anaerobe Mischflora zeigten. Eine mögliche Erklärung für den Nachweis ist ein Übertragungseffekt von der identifizierten Affenpockenvirus-DNA-positiven Zungenläsion. In diesem Fall dürfte die Viruslast in den Tonsillen viel geringer sein als in der Primärläsion. Interessanterweise waren die „cycle threshold“-Werte der untersuchten Proben vergleichbar, was darauf hindeutet, dass die nachgewiesene Affenpockenvirus-DNA tatsächlich aus dem Tonsillengewebe extrahiert und nicht über den Speichel aus der Zungenläsion eingeschwemmt wurde. In Tiermodellen wurde die Ausbreitung des Virus auf Tonsillen und Lymphknoten bereits vor dem Ausbruch des Exanthems beobachtet [9].

Obwohl wir über einen Einzelfall berichten, könnte dieser klinische Verlauf aufgrund der zunehmenden weltweiten Verbreitung in Zukunft häufiger auftreten als bisher beschrieben. Angemessene persönliche Schutzausrüstung und eine frühzeitige Diagnose sind für den Schutz des medizinischen Personals unerlässlich.

## Fazit für die Praxis

- Affenpocken erfordern eine rasche Diagnose, um eine weitere Übertragung von Mensch zu Mensch zu verhindern.
- Wichtig ist, dass sich eine Affenpocken-Infektion hinter einer vermeintlichen Mandelentzündung verstecken kann.
- Daher ist es wichtig, alle Angehörigen der Gesundheitsberufe zu sensibilisieren und nicht nur auf das typische Exanthem, sondern auch auf isolierte orale Läsionen zu achten, insbesondere bei Patienten, bei denen ein Risiko für Affenpocken-Infektionen besteht.
